# The spectrum and severity of psychopathological symptoms in previously healthy individuals who have had severe COVID-19 pneumonia

**DOI:** 10.1192/j.eurpsy.2021.755

**Published:** 2021-08-13

**Authors:** I. Belokrylov, N. Kotovskaya, N. Deryugina, A. Izyumov, K. Eruslanova, M. Balaeva

**Affiliations:** 1 Department Of Psychiatry And Medical Psychology, Peoples Friendship University of Russia (RUDN University), Moscow, Russian Federation; 2 Department Of Psychiatry And Medical Psychologi, Peoples Friendship University of Russia (RUDN University), Moscow, Russian Federation; 3 Department Of Math, Lomonosov Moscow State Univeristy, Moscow, Russian Federation; 4 Laboratory Of General Geriatrics And Neurogeriatrics, Pirogov RNMU – RCRCG, Moscow, Moscow, Russian Federation; 5 Laboratory Of Cardiovascular Aging, Pirogov RNMU – RCRCG, Moscow, Moscow, Russian Federation; 6 Department Of Aging Diseases, Pirogov RNMU – RCRCG, Moscow, Moscow, Russian Federation

## Abstract

**Introduction:**

The medical novelty of COVID-19 requires a comprehensive study of its impact on various areas of human health, including mental health.

**Objectives:**

To study the spectrum and severity of psychopathological disorders in previously healthy patients of different age groups who have had moderate and severe COVID-19 pneumonia.

**Methods:**

Immediately after stabilization of the physical condition, patients completed the Symptom Checklist-90-R, designed to assess 11 parameters: somatization (SOM), obsessive-compulsive (OS), interpersonal sensitivity (INT), depression (DEP), anxiety (ANX), hostility (HOS), phobic anxiety (PHOB), paranoid ideas (PAR), psychoticism (PSY). Patients with cognitive impairment were excluded.

**Results:**

The study involved 148 patients aged from 26 to 84 years. In the general sample, psychopathological symptoms were detected mainly on the SOM, DEP, ANX, HOS scales. To a lesser extent - on the INT and PAR scales; were practically not determined on the PSY and PHOB scales. Most of the symptoms are significantly more intense in patients over 46 years old (n = 129) compared with the younger population (<46 years old, n = 19). Older patients according to SOM revealed 1.23 points (IQR 0.5) versus 0.85 (IQR 0.7) among young people, DEP - 0.88 (IQR 0.44) vs. 0.47 (IQR 0.44), ANX - 0.66 (IQR 0.44) vs. 0.43 (IQR 0.29), OS - 0.55 (IQR 0.5) vs. 0.31 (IQR 0.25) and HOS - 0.46 (IQR 0.34) vs. 0.29 (IQR 0.09).

**Conclusions:**

Patients recovering from severe COVID-19 pneumonia require psychiatric evaluation and subsequent differentiated psychotherapeutic rehabilitation, especially for the age group over 46.

**Conflict of interest:**

No significant relationships.

